# Epidemiological Observations on Cryptosporidiosis in Diarrheic Goat Kids in Greece

**DOI:** 10.1155/2015/764193

**Published:** 2015-12-24

**Authors:** Nektarios D. Giadinis, Elias Papadopoulos, Shawkat Q. Lafi, Vasiliki Papanikolopoulou, Sofia Karanikola, Anastasia Diakou, Vergos Vergidis, Lihua Xiao, Evi Ioannidou, Harilaos Karatzias

**Affiliations:** ^1^Clinic of Farm Animals, Faculty of Veterinary Medicine, Aristotle University, 546 27 Thessaloniki, Greece; ^2^Laboratory of Parasitology and Parasitic Diseases, Faculty of Veterinary Medicine, Aristotle University, 541 24 Thessaloniki, Greece; ^3^Department of Pathology and Animal Health, Faculty of Veterinary Medicine, Jordan University of Science and Technology, P.O. Box 3030, Irbid, Jordan; ^4^National Veterinary Laboratory, 69100 Komotini, Greece; ^5^Division of Foodborne, Waterborne and Environmental Diseases, Centers for Disease Control and Prevention, Atlanta, GA, USA

## Abstract

This study aimed at investigating the occurrence of* Cryptosporidium* spp. in diarrheic goat kids in Greece and the risk factors associated with cryptosporidiosis. Altogether, 292 diarrheic 4–15-day-old goat kids from 54 dairy goat herds of Northern Greece were examined. Oocysts of* Cryptosporidium* spp. were detected in 223 of 292 (76.4%) goat kids and the intensity of infection was scored as “high” in 142 samples, “moderate” in 45 samples, and “low” in 36 samples. Larger herds (>200 animals) had higher infection rates than smaller ones, although this difference was not statistically significant. Significantly higher infection rates were observed in herds during late kidding season (1 January to 30 April) compared to the early one (1 September to 31 December). These results suggest that cryptosporidiosis is very common in diarrheic goat kids in Greece, especially in large herds during the late parturition season.

## 1. Introduction

*Cryptosporidium* spp. have a significant impact on health of neonatal 4–15-day-old goat kids, causing diarrhea, dehydration, and electrolyte imbalance. Cryptosporidiosis results in economic losses, because of the high morbidity and mortality rates, associated with the infection of this protozoan parasite. In addition, some of the* Cryptosporidium* isolates are zoonotic [1–4].

*Cryptosporidium* infection of diarrheic goat kids appears to be common in Southern Europe; many relative studies have been conducted in Turkey [5], Italy [6], Serbia [7], France [8, 9], Spain [10], and Cyprus [4]. Although cryptosporidiosis is an important cause of illness and mortality in goat kids, only one study has been conducted to date to investigate the risk factors involved in the acquisition of infections in this animal species [8], while the rest are based on simple clinical observations [4, 5, 11].

In Greece, studies of neonatal cryptosporidiosis have been conducted to date in dairy calves [12] and lambs [13], but a similar study has not been conducted in goat kids. This study aimed at investigating the occurrence of* Cryptosporidium* spp. in diarrheic goat kids in Greece and at providing some epidemiological data that could contribute to the better understanding of cryptosporidiosis.

## 2. Materials and Methods

This study was conducted in Northern Greece from October 2011 to May 2013 in 54 goat herds (22,890 adult animals) with over 10% incidence of diarrhea in 4–15-day-old goat kids. Younger and older animals were not included in this study. The herds included in this report consisted of local dairy crossbreeds maintained under the semi-intensive feeding system, which is the most common one in the country. They were regularly dewormed and vaccinated against clostridiosis. The selected herds had not received any anticryptosporidial medication prior to sampling. Each herd or animal was sampled only once.

The Ambulatory Clinic of the Faculty of Veterinary Medicine of Aristotle University visited the aforementioned goat herds. A complete history of the case was taken and rectal fecal samples were collected from at least 4 goat kids with diarrhea per herd. The samples were transferred to the Laboratory of Parasitology and Parasitic Diseases at the Faculty of Veterinary Medicine of Aristotle University and examined immediately.

The fecal samples were diluted with tap water and passed through a sieve (no. 150) in a centrifuge tube, centrifuged at 500 g for 3 min and smears of 20 *μ*L of the sediment were dried, stained using the acid-fast Ziehl-Neelsen technique [14], and examined at 1000x magnification for* Cryptosporidium* spp. oocysts. The intensity of the infection was estimated semiquantitatively according to the average number of oocysts in 10 random fields. It was scored as light (<5 oocysts/10 fields), moderate (5–10 oocysts/10 fields), and high (>10 oocysts/10 fields). If no oocysts were detected, it was scored as negative.

Risk factors were also investigated and data on herd size (“large herds” ≥200 animals or “small herds” <200 animals) and kidding season were collected. Goat kids born from 1 September to 31 December were called “early,” while those born from 1 January to 30 April were called “late.”

Data were collected and entered in a data sheet and analysed using IBM SPSS 20.0 2011 software for Windows (IBM SPSS Corp., Armonk, NY, USA). Chi-square test (or Fisher's Exact Test) was used to determine the relationships between studied risk factors and sample positivity in goat herds. A *P* value ≤ 0.05 was considered statistically significant.

## 3. Results

Eight of the 54 goat herds had less than 200 adult animals, while the remaining 46 herds had ≥200 adult animals. Forty-five of the herds (83.3%) were found positive, with at least one animal infected with* Cryptosporidium* spp.: 5 in herds with <200 animals and 40 in herds with ≥200 animals. The herd size did not influence significantly (*P* > 0.05) the infection with* Cryptosporidium* spp. Most of the affected goat kids belonged to “late” kidding herds. In fact, 37 of the 45 positive herds (82.2%) were from “late” kidding and the remaining 8 (17.8%) were from “early” kidding. The difference between “early” and “late” herds was statistically significant (*P* < 0.01). All of the data in this paragraph are listed in Table 1.

On kid level, the occurrence rate of cryptosporidiosis was 76.4% (of the 292 goat kids that participated in the current study, 223 or 76.4% were infected with* Cryptosporidium* spp.). Regarding the intensity of the infection, 142 samples (48.63%) were scored as “high,” 45 (15.41%) were scored as “moderate,” and 36 (12.33%) were scored as “low,” while the remaining 69 samples (23.63%) were “negative.”

## 4. Discussion

Diarrhoea syndrome of neonatal goat kids causes significant losses worldwide and also in Greece [2, 15]. Cryptosporidiosis seems to be one of the most important causes of this syndrome in Greece and other countries [1, 16, 17].

In Greece, the contribution of* Cryptosporidium* spp. to neonatal goat kid diarrhoea and mortality has already been noticed in small number of goat herds [1, 18]. However, a systematic study has not been conducted to investigate its prevalence in goat herds with high neonatal goat kid diarrhoea incidence; the existing studies have investigated its prevalence in adult goats or herds with nonremarkable incidence of diarrhoea in neonatal goat kids [19–21].

The contribution of cryptosporidiosis in diarrhoea syndrome at the age of 4–15 days has been assessed to be 46% in Greek dairy calves [12] and 29% in lambs [13]. In the present study it was found that 76.4% of the diarrheic goat kids were positive for* Cryptosporidium* spp., as well as the 83.3% of the 54 examined goat herds. In fact, the data show that the problem of cryptosporidiosis seems to be much more common in the goat herds than in the sheep or dairy cattle in Greece [12, 13]. In a similar study in Cyprus the percentages were similarly high on both sheep and goat farms [4], although the percentage of positive goat herds was slightly higher than that in Greece (89.3% in Cyprus and 83.3% in Greece). Also, a high occurrence of* Cryptosporidium* infection has been found in diarrheic goat kids in a large French study by Delafosse and coworkers [8].

Herds with high animal stock were more likely to have* Cryptosporidium* spp. infection and cryptosporidiosis, although this difference was not statistically significant. In a similar study conducted in Cyprus the difference was found to be statistically significant [4], although the number of goat herds examined was lower. However, large herd size can be a risk factor for increased* Cryptosporidium* spp. infection, as it has been already shown in cattle [22] and sheep [4, 23, 24]. The increased herd size can affect the prevalence of* Cryptosporidium* spp. infection, because of inadequate management of these herds and close contact among susceptible animals [4].

“Late” kidding seems to be a factor that increases the possibility of clinical cryptosporidiosis. This finding confirms previous findings in goat kids [4, 8] and in lambs [4, 23]. The Greek goat-keepers usually clean animal premises once every year (in the summer); thus when the kidding season is extended the parasite contamination increases in the premises [4, 13]. This seems to be the most probable explanation for this condition, although a systematic research is necessary.

The majority of the examined diarrheic goat kids (48.63%) had a heavy* Cryptosporidium* spp. infection. In other studies in goats [8] and cattle [25], it was found that heavily infected goat kids and calves suffered from severe diarrhoea. It has also been previously reported that lambs or goat kids heavily infected with* Cryptosporidium* spp. can suffer from fatal diarrhoea with cryptosporidiosis being the sole cause or the predisposing factor for other pathological entities [1, 26, 27].

Furthermore, in neighbouring countries of Greece, the role of cryptosporidiosis in goat kids as well as in other animal species and human health has also been assessed [5, 7, 28–30]. Additionally, in Bulgaria, a study has been conducted for the presence of* Cryptosporidium* spp. in different water supplies [31]. Therefore, it seems that cryptosporidiosis is a common cause of diarrhoea in newborn goat kids in Greece and other countries of Balkan Peninsula. This could be due to the similar climatic and husbandry conditions, and definitely more extensive epidemiological studies in this area are necessary for the effective protection of animal and public health.

Conclusively, cryptosporidiosis is very common in diarrheic goat kids in Greece, especially for the large herds as well as for “late” kidding animals. This parasitic infection may pose a significant health and production issue in goat herds and a public health hazard.

## Figures and Tables

**Table 1:** Prevalence (%) of *Cryptosporidium* spp. infection in goat herds related to their size and time of kidding.

Goat herds	Herd size		* *Time of kidding		Total
	≥200 animals	<200 animals	Late	Early	
Infected	40	5	37	8	45 (83.3%)
Not infected	6	3	2	7	9 (16.7%)

Total	46	8	39	15	54
